# Review article inferior vena cava thrombosis: a case series of patients observed in Taiwan and literature review

**DOI:** 10.1186/s12959-021-00296-5

**Published:** 2021-06-22

**Authors:** Hsuan-Yu Lin, Ching-Yeh Lin, Ming-Ching Shen

**Affiliations:** 1 Division of Hematology/Oncology, Changhua Christian Hospital, Nanshiao St 135, Changhua City, Changhua County, Taiwan; 2 Department of Laboratory Medicine, Department of Internal Medicine, National Taiwan University Hospital, Taipei, Taiwan

**Keywords:** Inferior vena cava, Venous thromboembolism, Pulmonary embolism, Taiwan, Vena cava filters, Post-thrombotic syndrome

## Abstract

Inferior vena cava thrombosis (IVCT) is rare and can be under-recognized. However, the associated complications and mortality may be severe. We report the first case series of IVCT observed in Taiwan with a brief literature review. Eight Taiwanese patients with IVCT between May 2012 and December 2019 were enrolled in this study. Deep venous thrombosis (DVT, 8/8) and pulmonary embolism (5/8) were reported. Various risk factors were identified, including an unretrieved inferior vena cava (IVC) filter, pregnancy, surgery, presence of lupus of anticoagulants, essential thrombocythemia, antithrombin deficiency, and hemoglobin H disease. Of note, four of our patients experienced complete IVC thrombosis with bilateral lower extremity swelling (due to DVT) and abdominal wall superficial venous dilatation, while four other patients presented with partial IVCT and unilateral DVT. The etiology, clinical characteristics, presentations, diagnosis, and treatment of IVCT were reviewed.

## Background

Inferior vena cava thrombosis (IVCT) is a clinically rare condition [1]. According to the United States National Hospital Discharge Survey, vena cava thrombosis (presumed to be predominantly IVCT) accounted for only 1.3 % of all hospitalized patients who were diagnosed with venous thrombosis between 1979 and 2005 [2]. In Asia, a low incidence of IVCT is plausible, since a lower risk and incidence of venous thromboembolism (VTE) in ethnic Asian populations have been demonstrated [3]. However, the true incidence of IVCT could be underestimated due to a lack of standardized detection methods and insufficient clinical awareness. Therefore, IVCT may be an under-recognized condition.

Although IVCT is not commonly identified, the associated acute or chronic complications are significant and alarming. These include post-thrombotic syndrome (PTS, 90 %), venous claudication (45 %), pulmonary embolism (PE, 30 %), and venous ulceration (15 %) [4]. Furthermore, the mortality rate of IVCT has been reported to be two-fold higher than that of deep vein thromboses (DVT) confined to the lower extremities. This implies that the consequences of IVCT can be serious [5].

Since 2010, IVCT has emerged as a significant issue. The United States Food and Drug Administration (FDA) released an inferior vena cava (IVC) filter-related device safety communication that indicated a potential risk of IVC filter-related complications [6], including IVCT. The FDA also recommended that the implanting physicians and clinicians responsible for the ongoing care of patients with retrievable IVC filters should consider removing the filter as soon as protection from PE is no longer required [7]. This recommendation was made to reduce the risks associated with chronic indwelling IVC filters.

In this study, we collected data from eight Taiwanese patients from two medical centers diagnosed with IVCT, including two patients who presented with complications after an IVC filter was placed and unretrieved. Their primary clinical information, coexisting thrombotic risk factors, additional sites of venous thrombosis, and treatment outcomes were reported. In addition, a brief review of IVCT from the literature would be presented.

## Case series

### Patients and methods

Eight patients diagnosed with IVCT between May 2012 and December 2019 at Changhua Christian Hospital and National Taiwan University Hospital were included in this study. All laboratory tests and radiological imaging examinations were performed as part of routine clinical evaluations. All of our eight patients with IVCT were diagnosed by abdominal computed tomography, while their DVT were detected by compression ultrasonography. Patient demographics, presenting characteristics, additional sites of venous thrombosis, location and extent of the IVCT, treatment outcome, and related adverse events were investigated. All patients provided informed consent to participate in this study, and the study was conducted in accordance with the Declaration of Helsinki.

### Results

In our report, out of the eight patients with IVCT, three were men and five were women. Their ages ranged from 30 to 54 years (median age, 35.5 years). DVT associated with unilateral lower extremity swelling was detected in four patients (in the left lower extremity in two patients, and in the right lower extremity in two patients). Bilateral lower extremity swelling was detected in the remaining four patients who also presented with dilatation of the superficial veins of the abdominal wall. Collateral circulation within the abdomen was observed in five patients. Clinical presentations, extent of IVCT, and risk factors are summarized in Table 1.

**Table 1:** Clinical presentations and characteristics of eight Taiwanese patients with inferior vena cava thrombosis

Patient no.	Age (year)	Sex	Main clinical presentation		Site of thrombosis					Collaterals inside the abdomen	Risk factors
			Swelling of LE	Dilatation of SAV	IVC	IVs	PDV	DDV	PE		
1	30	F	L + R	Present	IHCT	L + R	L + R	L + R	(+)	Present	ET + pregnancy
2	35	M	L + R	Present	IHCT	L + R	L + R	(-)	(+)	Present	LA
3	32	F	L	None	IRPT	(-)	L	L	(-)	None	LA + pregnancy + surgery
4	36	F	L	None	IRPT	L	L	(-)	(-)	None	Surgery
5	54	F	L + R	Present	IRCT	L + R	L + R	L + R	(-)	Present	Unknown
6	32	M	R	None	IRPT	R	R	R	(+)	None	AT deficiency
7	38	M	L + R	Present	IRCT	L + R	L + R	(-)	(+)	Present	LA + IVC filter
8	46	F	R	None	IRPT	(-)	R	R	(+)	Present	Hb H disease + pregnancy + IVC filter

Clinical pictures of patients with inferior vena cava thrombosis evaluated at Changhua Christian Hospital and National Taiwan University Hospital

*LE* lower extremity, *SAV* superficial abdominal wall vein, *IVC* inferior vena cava, *IVs* iliac veins, *PDV* proximal deep vein, *DDV* distal deep vein, *PE* pulmonary embolism, *F* female, *M* male, *R* right, *L* left, *IHCT* infrahepatic complete thrombosis, *IRCT* infrarenal complete thrombosis, *IRPT* infrarenal partial

No congenital IVC anomalies were detected in our cohort. All of our eight IVCT patients had DVT, while five (62.5 %) also had PE. Prior placement of an IVC filter was found to be an important risk factor for IVCT (25 %). Other VTE predisposing risk factors, including pregnancy (37.5 %), presence of lupus anticoagulants (37.5 %), surgery (25 %), essential thrombocythemia (ET, 12.5 %), antithrombin deficiency (12.5 %), and hemoglobin H disease (12.5 %), were observed. While three of our patients exhibited lupus anticoagulants, no one expressed anti-cardiolipin antibodies. One patient presented with no known risk factors. Four patients had more than one prothrombotic risk factor. No patient had experienced cancer or Budd-Chiari syndrome (BCS) in our study.

All patients with IVCT in this study received anticoagulants. One patient (patient 6) underwent catheter-directed mechanical thrombectomy through the right lower extremity route. However, the procedure failed as the catheter could not pass through the femoral vein thrombosis. The decision regarding which investigational methods were to be used for IVCT treatment was made by the attending physician. One patient (patient 8) developed PTS (12.5 %). No IVCT-related mortality was reported in our series.

### Case presentation

**Fig. 1:**
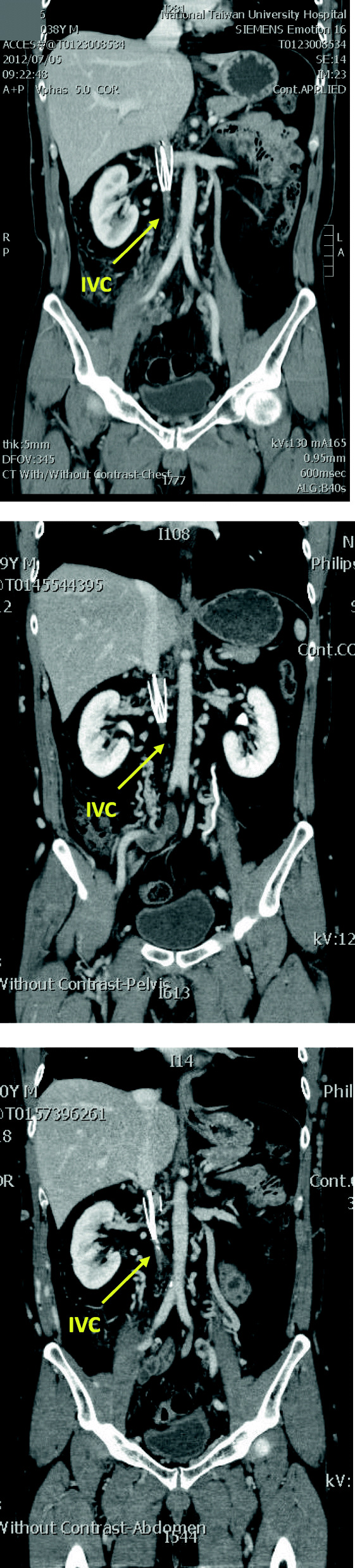
Bilateral lower extremity DVT and IVC thrombosis occurred in a 38-year-old man. (**A**) Computed tomography demonstrated a filter bearing IVC thrombosis. In the follow-up at 21 months (**B**) and 33 months (**C**) later, computed tomography revealed a residual thrombosis. DVT, deep venous thrombosis; IVC, inferior vena cava

The clinical presentation and characteristics of one patient with IVCT in this study are briefly described below: Patient 7 was a 38-year-old man. He experienced VTE with repeated episodes of painful swelling in his right lower extremity, accompanied by upper back pain and cough. The patient was diagnosed with DVT and PE. Anticoagulant treatment was initiated. An IVC filter was placed after the second VTE event. However, 6 months after filter placement, the patient had swellings in both lower extremities. The superficial veins of the abdominal and chest walls were dilated. Filter retrieval was attempted twice but was unsuccessful. A positive result for lupus anticoagulant was the only significant finding from thrombotic screening tests. Compression ultrasonography revealed partial thrombosis in the common femoral veins, superficial femoral veins, and popliteal veins of both lower extremities. Computed tomography revealed complete thrombosis in the infrarenal IVC along with the presence of an IVC filter and blood clots within both iliac veins. Collateral circulation was also observed (Fig. 1).

## Literature review

To offer a thorough understanding of IVCT, a brief review from the literature is described below. Readers are also encouraged to read other comprehensive review articles [1, 4, 8]. In addition, the findings of several previous reports of IVCT as well as our study were summarized in Table 2.

**Table 2:** Summary of studies on inferior vena cava thrombosis

	Stein et al. [2]	Linnemann et al. [9]	Linnemann et al. [10]	Kraft et al. [11]	Teter et al. [12]	Lin et al. ^a^
Study design	Retrospective, NHDS database	Retrospective, MAISTHRO database			Retrospective, single institute	Retrospective, two institutes
Study periods	1979 to 2005	Mar 2000 to Aug 2006	Mar 2000 to Nov 2007	Mar 2000 to Feb 2010	Jan 2013 to July 2016	May 2012 to Dec 2019
Reported patient numbers	99,000 VCT patients	53 IVCT patients	60 IVCT patients, only 40 patients enrolled	141 IVCT patients	41 IVCT patients	8 IVCT patients
Study areas	USA 50 states	Germany			New York	Taiwan
Proportion of VTE (IVCT/VTE)	99,000/ 7,902,000 (1.3 %)	53/1170 (3 %)	60/1421 (4.2 %)	141/1500 (9.4 %)	NA	NA
Gender (F/M) n (%)	50,400 (51.1 %) /48,200 (48.9 %)	35 (66 %)/ 18 (34 %)	25 (63 %) / 15 (37 %)	81 (57 %)/ 60 (43 %)	21 (51.2 %) / 20 (48.8 %)	5 (62.5 %)/ 3 (37.5 %)
Age (median)	0–70+ (NA)	12–79 (35.6)	12–38 (34)	12–85 (47)	15–91 (61)	30–54 (35.5)
Clinical characteristics						
Location and extent of IVCT, n (%)	NA	Suprarenal segment, 3 (6 %); infrarenal segment, 50 (94 %)	NA	Hepatic segment,12 (8.5 %); suprarenal segment, 12 (8.5 %); infrarenal segment, 139 (98.6 %)	NA	Infrahepatic segment, 2 (25 %); infrarenal segment, 6 (75 %)
Isolated IVCT, n (%)	77,000 (78 %)	4 (7.6 %)	1 (2.5 %)	NA	NA	0 (0 %)
Thrombosis involving other venous segments, n (%)	22,000 (22 %) coexisted with DVT	Hepatic veins, 1 (1.9 %); renal veins, 2 (3.8 %); iliac veins, 46 (87 %); femoral veins, 42 (79 %); calf veins, 27 (51 %)	Hepatic veins, 1 (2.5 %); iliac veins, 37 (93 %); femoropopliteal veins, 30 (75 %); calf veins, 14 (35 %)	Right atrium, 10 (7.1 %); hepatic veins, 6 (4.3 %); renal veins, 24 (17 %); iliac veins, 94 (66.7 %); femoral veins, 69 (48.9 %); popliteal veins, 49 (34.8 %); calf veins, 31 (22 %)	NA	8 (100 %) with DVT; proximal veins, 8 (100 %); distal veins 5 (62.5 %)
Bilateral lower extremities DVT, n (%)	NA	13 (28.3 %)	NA	NA	NA	4 (50 %)
Bilateral DVT with dilatation of the superficial veins of the abdominal wall, n (%)	NA	NA	NA	NA	NA	4 (50 %)
Collaterals inside the abdomen, n (%)	NA	NA	NA	NA	NA	5 (62.5 %)
PE, n (%)	9400 (12 %)	17 (32.1 %)	NA	38 (27.0 %)	11 (26.8 %); 7 with IVC filters	5 (62.5 %); 2 with IVC filters

*NHDS* National Hospital Discharge Survey, *MAISTHRO* MAIn-ISar-THROmbose-Register, *VCT* vena cava thrombosis, *IVCT* inferior vena cava thrombosis, *IVC* inferior vena cava, *NA* not available, *VTE* venous thromboembolism, *F* female, *M* male, *DVT* deep venous thrombosis, *PE* pulmonary embolism

^a^ Present study

### Etiology

IVCT accounts for a minority of VTE cases, with a reported incidence of 4–15 % in patients with DVT [13]. In accordance with VTE, IVCT could be caused by congenital or acquired abdominal pathophysiological changes with venous blood flow turbulence or stasis consequences, combined with the presence of various prothrombotic factors predisposing to VTE. Idiopathic/primary IVCT occurs without identifiable cause. It was reported to be associated with risk factors similar to those predisposing to VTE, while hypertension or metabolic syndrome-related VTE might be involved [8]. Provoked/secondary IVCT often refers to the thrombus generated from the precedent lower extremity DVT with propagation or extension into the IVC, while cancer, surgery, infection, and oral contraceptive-related VTEs are also classified as provoked and secondary [8].

Various VTE risk factors of IVCT patients from a database registration had been investigated [9–11]. Local problems such as IVC anomalies (11.3 %) and external venous compression (11.3 %), malignancy (17.0 %) and the presence of lupus anticoagulants (10.9 %), contribute to the risk of IVC thrombosis [9]. In addition, hereditary thrombophilia, hormonal treatment in woman, and previous surgery were also reported to be common risk factors of thrombosis involving IVC [10]. Table 3 summarized the etiology reported from several previous studies.

**Table 3:** Summary of etiology reported from studies on inferior vena cava thrombosis

	Stein et al. [2]	Linnemann et al. [9]	Linnemann et al. [10]	Kraft et al. [11]	Teter et al. [12]	Lin et al. ^a^
Primary/unprovoked IVCT	NA	NA	16 (40 %)	32 (22.7 %)	NA	1 (12.5 %)
Congenital IVC anomalies, n (%)	NA	6 (11.3 %)	NA	18 (12.8 %)	NA	0 (0 %)
External compression, n (%)	NA	6 (11.3 %)	NA	9/55 (16.4 %) of reported malignancy related IVCT	NA	0 (0 %)
Malignancy, n (%), most common cancer types	37,000 (37.4 %), kidney; trachea, bronchus, and lung	9 (17.0 %), lung, breast, kidney, bladder, ovary, brain	6 (15 %), NA	55 (39 %), renal, ovary, testes, breast, lymphoma	17 (41.5 %), NA	0 (0 %)
Hereditary thrombophilia, n (%)	NA	28 (52.8 %)	27 (51 %)	37 (38.9 %)	4 (9.8 %)	1 (12.5 %)
Antithrombin deficiency, n (%)	NA	2 (3.8 %)	NA	2 (1.8 %)	NA	1 (12.5 %)
Lupus anticoagulants /APS, n (%)	NA	5 (10.9 %)	3 (6 %)	6 (5.8 %)	NA	3 (37.5 %)
Anticardiolipin antibody, n (%)	NA	2 (3.8 %)	NA	NA	NA	0 (0 %)
Pregnancy, n (%)	NA	NA	1(4 %)	2 (2.5 %)	NA	2 (25 %)
Obesity, n (%)	NA	7 (14.9 %)	NA	NA	11 (26.8 %)	0 (0 %)
Surgery, n (%)	NA	11 (20.8 %)	6 (15 %)	17 (12.1 %)	NA	2 (25 %)
IVC filters, n (%)	NA	NA	NA	NA	18 (43.9 %)	2 (25 %)
Oral contraceptives / hormone treatment, n (%)	NA	13 (37.1 %)	12 (48 %)	25 (30.9 %)	3 (7.3 %)	0 (0 %)
Other risk factors, n (%)	NA	Family history of DVT, 6 (12.7 %); inflammatory disease, 8 (15.1 %)	Inflammatory disease, 5 (13 %); immobilization 2 (5 %); MPN 0 (0 %); JAK-2 V617F mutation 0 (0 %)	Inflammatory disease, 23 (16.3 %); risk-associated DVT, 109 (77.3 %)	History of prior DVT, 25 (61.0 %); smoking, 18 (43.9 %)	ET, 1 (12.5 %); hemoglobin H disease, 1 (12.5 %)

*IVCT* inferior vena cava thrombosis, *NA *not available, *IVC *inferior vena cava, *VTE *venous thromboembolism, *APS* antiphopholipid antibody syndrome, *MPN* myeloproliferative neoplasm, *DVT* deep venous thrombosis, *ET* essential thrombocythemia

^a^ Present Study

#### Congenital anomalies of inferior vena cava

Congenital IVC anomalies are infrequent, with an estimated incidence of 0.3–0.6 % in the general population [14]. Congenital IVC anomalies include segmental hypoplasia or aplasia of the IVC and a venous aneurysm, and are usually classified into the following three main anatomic configurations [15, 16]: (a) infrarenal type (duplicate IVC, persistent left side IVC, preaortic IVC, absence of the infrarenal IVC), (b) renal type (accessory left renal vein, retroaortic and circumaortic left renal vein), and (c) suprarenal type (absence of the hepatic IVC with azygous continuation, congenital caval stenosis or atresis, IVC membrances). Each configuration with resultant turbulent blood flow has the capacity to lead to the formation of a thrombus within IVC. Due to well-developed collaterals, congenital IVC anomalies rarely manifest as symptoms and are often incidental imaging findings. Thromboembolic symptoms might be caused by the involvement of venous collaterals, frequently deep pelvic veins or common iliac veins. In the presence of caval aberrancy, bilateral iliofemoral thrombosis was found in 66–75 % of patients [17, 18], while among all of the lower limb DVT patients, bilateral iliofemoral thromboses are reported to be uncommon, accounting for fewer than 10 % of cases [19]. With a reported incidence of 60–80 %, individuals with congenital abnormalities of the IVC are more likely to develop VTE or IVCT, particularly in younger patients [17, 18, 20]. A systemic review reported that congenital IVC anomalies, with a weighted prevalence of 6.8 %, could lead to a 50- to 100-fold increase in the risk of DVT [21].

#### Tumors

While the risk of VTE in cancer patients is 7-fold higher than that in non-cancer populations [22], malignancy has been reported to account for 37.5 % of IVCT cases in a large observational study, demonstrating its close relationship with IVCT [2]. Diverse cancer types have been reported in patients with IVCT. The most common type is renal cell carcinoma (38 %), followed by other genitourinary tract cancer (25 %) [11]. Established mechanisms including external compression of the IVC by tumor masses, progression of malignancy into the IVC (tumor thrombus), and malignancy-related hypercoagulability, have all been highlighted. It is reported that malignancy-related IVCT more frequently involve the suprarenal and hepatic segments of the IVC and extend more often into the right atrium than does IVCT [11]. In addition, retroperitoneal leiomyosarcoma [23], adrenal cortical carcinoma [24], and renal angiomyolipoma [25] have all been shown to be associated with IVCT. Furthermore, antineoplastic therapy, such as surgery or chemotherapy, could present prothrombotic risks contributing to VTE.

#### Compression/outflow obstruction

Direct extrinsic compression or venous outflow obstruction can trigger thrombogenesis within the IVC. Extrinsic compression may also be caused by non-tumor sources, such as aortic aneurysm, retroperitoneal fibrosis, or retroperitoneal hematoma [8].

##### Budd-Chiari syndrome

Budd-Chiari syndrome (BCS) is characterized by the manifestations caused by hepatic venous outflow obstruction. The obstruction may occur secondarily to extrinsic compression of the hepatic vein or hepatic segment of the IVC, such as the mass effects from hepatocellular carcinoma or other lesions of the liver. Other etiology of BCS includes myeloproliferative neoplasms (MPNs), hereditary thrombophilia, pregnancy, paroxysmal nocturnal hemoglobinuria, or oral contraceptives. Additionally, membranous obstruction of the IVC at its hepatic portion, also known as obliterative hepatocavopathy, has been reported to be another etiology that has prevailed in Asia [26, 27]. Distinct pathogenesis of the “membrane” formation within the IVC at the level of the diaphragm with or without hepatic vein involvement has been proposed to be the consequences of recurrent thrombosis [26, 27]. Hepatic vein thrombosis may present as thrombogenesis provoked by a venostasis scenario, or as direct extension from a generated IVC thrombus to the intrahepatic vessels. On the other hand, IVCT may develop following the thrombotic occlusion of hepatic veins, which indicates BCS. Among patients of BCS, 20 % are estimated to be associated with IVCT [28].

##### May-Thurner syndrome

May-Thurner syndrome is noted for symptomatic left lower extremity swelling with increased risk of DVT formation. It is caused by compression of the left common iliac vein from the overlying right common iliac artery [29]. Chronic May-Thurner syndrome coexisted with DVT may lead to thrombus propagation to the IVC.

##### Pregnancy

Pregnancy was reported to be occurred in 11.4 % IVCT patients [9]. In late pregnancy, the distended uterus can compress the IVC particularly in the supine position, which leads to decreased cardiac output, hypotension, venous congestion, and stasis [30, 31], thereby increasing the risk of IVCT. The risk of VTE is increased 5- to 10-fold in pregnancy and the puerperium [32, 33], complicating 1 in 1,000 deliveries [34]. VTE is a leading cause of maternal mortality in the developed world [35]. Pregnancy-associated VTE reflects the hypercoagulability that has evolved to protect women from hemorrhage at the time of childbirth or miscarriage [36]. The risk of VTE is highest immediately after delivery, specifically 3 to 6 weeks postpartum, after which the risk declines [37].

##### Obesity

Obesity has been reported to be a risk factor for IVCT [38]. An increased pressure gradient between the thoracic and abdominal vena cava was found to be associated with a high body mass index (> 30 kg/m^2^), while two obese patients presented recurrent thrombosis [38].

##### Trauma

Trauma-related IVCT were caused by a combination of various mechanisms [39]. Transmural laceration of the vena cava secondary to crushing forces, with formation of a pericaval hematoma, can compress and narrow the vena cava and cause venous stasis. Furthermore, endothelial injury of the venous wall may contribute to caval mural thrombosis [40]. Hepatic vein thrombosis with extension to IVC may occur if hepatic parenchyma is injured. In addition, hypercoagulability with suppression of fibrinolysis characterizes the physiological hemostatic responses after trauma while promotes IVCT.

#### Provoked/secondary IVCT without outflow obstruction

##### Lupus anticoagulants/anti-cardiolipin antibodies

The presence of lupus anticoagulants and antiphospholipid syndrome have been revealed to be associated with IVCT [9]. Among patients with IVCT, 10.9 and 3.8 % were reported to exhibit lupus anticoagulants and anti-cardiolipin antibodies, respectively [9]. Two studies reviewed computed tomography of patients with antiphopholipid syndrome and reported IVCT in 8/12 and 10/42 patients undergoing CT scan for suspected major abdominal vascular occlusion.[41, 42].

##### Inferior vena cava filters and others

Iatrogenic causes, such as an IVC filter, were found to be strongly associated with IVCT. The incidence of permanent filter-associated IVCT is approximately 13 % after 8 years of follow-up [43]. Other etiologies associated with IVCT have been disclosed, including hereditary thrombophilia, paroxysmal nocturnal hemoglobinuria [44], pancreatitis [45], inflammatory disorders [46], oral contraceptives or hormonal replacement therapy [47], and coronavirus-2019 (COVID19) infection [48], to mention a few. The risk of VTE is found to be increased significantly in the hospitalized COVID-19 patients, with an estimated incidence of 17 % (12.1 % DVT and 7.1 % PE) [49]. Established mechanisms of VTE associated with COVID-19 include a hyperinflammatory response, platelet activation, and triggering of the coagulation cascade [50].

### Diagnosis

Diagnosis of IVCT remains challenging at clinical presentation, which depended on the acuity level, extent of the thrombus, consequences of cava and/or splanchnic veins occlusion, and other accompanying VTE if present. Typical clinical features of IVCT include bilateral lower extremity DVT, scrotal swelling, unexplained back pain, pelvic pain, and in some cases, acute renal failure [51–54]. IVCT can present differently from lower-extremity DVT when IVCT also involves renal veins or hepatic veins. Renal vein involvement may result in flank pain and hematuria, whereas oliguria, anuria or uremia-related symptoms may indicate bilateral renal vein thrombosis. Hepatic vein involvement would compromise liver venous outflow, causing hepatic congestion, formation of ascites, and portal vein thrombosis. Chest pain and shortness of breath imply PE, which has been reported in 12 % of patients with IVCT [2]. The following high risk features also strongly suggest to the diagnosis of IVCT, including the presence of an unretrieved IVC filter, iliofemoral DVT, bilateral DVT, unexplained new back pain or renal failure, known congenital IVC anomalies, severe PTS, renal cell carcinoma, and BCS [4]. Acute thrombosis at IVC with both iliofemoral veins involvement without collateral venous network may lead into phlegmasia cerulean dolen, which manifests with cyanosis and venous gangrene changes. Coagulation laboratory tests incorporating D-dimer, VTE probability evaluations, and thrombophilia screening would be valuable for the diagnosis of IVCT as well as DVT.

### Imaging assessment

Multiple radiological modalities can be used to diagnose IVCT, including sonography, computed tomography, magnetic resonance imaging, and transcatheter venography. Sonography provides an accurate non-invasive evaluation and is often the first-line modality in investigating patients with lower extremity symptoms. Sonographic signs of IVCT include a monophasic Doppler waveform that does not synchronize with aspiratory rhythms and a “choppy” sign indicating increased blood velocity [55]. However, overlying bowel gas or body habitus can limit evaluations made by sonography. Contrast enhanced computed tomography with advances in 3D reconstruction allows clear depictions of the IVC structure and identifies the malignant nature of the thrombus [56]. Magnetic resonance imaging has advantages in delineating the presence, size and location of IVCT while avoiding radiation [57]. Catheterization of the IVC and hepatic veins can demonstrate patency or occlusion of the venous orifices.

### Treatment

The objectives of IVCT treatment include reducing the risk of PE, decreasing chronic complications such as PTS, and decreasing venous insufficiency and related symptoms. The cornerstone of IVCT management consists of in-time administration of anticoagulants, if not contraindicated. Unfractionated or low-molecular-weight heparin is commonly used, followed by bridging to oral anticoagulants such as warfarin. Novel direct oral anticoagulant agents may be a reasonable alternative. While some may respond to anticoagulation only, up to 40 % VTE patients reported thrombus propagation despite anticoagulation [58]. Additional treatment options depend on the acuity and severity of the thrombosis. Catheter-directed thrombolysis (CDT) alone or combined with pharmacomechanical thrombectomy have the advantages of rapid direct thrombolytic effects, which may reduce the risk of PTS complication for those with acute IVCT without high risk of bleeding [59], although not influencing the mortality significantly [60]. Urgent CDT is recommended for those patients with severe acute DVT associated with limb-threatening compromised or worsening IVCT despite anticoagulation [61]. The limitation of CDT is the potential increased risks of bleeding. Percutaneous transluminal angioplasty with a stent may be a reasonable choice for chronic thromobosis [62].

## Discussion

This study is the first report that has examined IVCT presentation and characteristics in Taiwanese patients, amid the paucity of academic literature on the subject of IVCT. Our goals are to enhance and acknowledge awareness of the rare but liable to be neglected clinical conditions.

Clearly, this study has certain limitations. One of the major drawbacks was selective bias, since the information was gathered from limited patients in two hemophilia treatment and thrombosis centers within affiliated medical institutes in Taiwan. In addition, the collected information from a small number of patients was insufficient to reveal the overall picture of IVCT. Still, more in-depth information about IVCT as well as the results of large-scale investigations such as the SIVECT (Study on Inferior Vena Cava Thrombosis) [63] are eagerly awaited. Nevertheless, our study has revealed several interesting findings that should be addressed.

First, our study highlighted that complete IVC thrombosis with bilateral lower extremity swelling (due to DVT) and abdominal wall superficial venous dilatation were present in four of our patients. This peculiar presentation can be regarded as a pathognomonic manifestation of complete IVCT, although it has been reported in only half of all IVCT patients [1, 51]. It is plausible that once bilateral venous circulation from the lower extremities was compromised by complete IVC thrombosis, collateral circulation would ensue with resultant manifestations of superficial venous dilatation at the abdominal walls. This explanation is supported by the evidence that four partial IVCT patients in our study coexisted with only unilateral DVT.

In addition, all IVCT patients were found to have DVT in our study. The association between IVCT and VTE has been previously described. Congenital IVC anomalies or venous outflow obstruction/compression scenarios, such as BCS, have the capacity to elicit IVCT without precedent DVT, although not all of the precipitating conditions associated with IVCT were identified in our case series. Moreover, the thrombus formed from precedent DVT could propagate or extend into the IVC, wherein various known VTE risk factors could also contribute to IVCT.

Several thromboembolic risk factors were identified in our study. Notably, one of our patients with IVCT was found to have hemoglobin H disease, along with other risk factors, including pregnancy and an IVC filter. Hemoglobin H disease, also known as α-thalassemia intermedia, has been identified as a hypercoagulable state with the demonstration of elevated thrombotic biomarkers [64, 65]. Abnormalities in pathologic red blood cells, activated platelets, endothelial damage, and splenectomy are established mechanisms of thalassemia that could contribute to VTE [66]. Although the association between thalassemia and IVCT has been reported sporadically [67], our findings suggest that the underlying hypercoagulable state of thalassemia may contribute to the development of IVCT, as well as other reported clinical thrombotic events.

One patient had ET. Venous thromboembolism and arterial thrombosis are the most common causes of morbidity and mortality in patients with MPNs, including ET [68]. Notably, thrombosis within the splanchnic or cerebral veins is a hallmark manifestation in patients with MPN [69]. The mechanisms of thrombotic potentials in MPN patients have been elaborated. They include increased blood counts, chronic inflammation, JAK-2 mutated endothelium, and aberrant platelet-neutrophil interactions [70]. Advanced age (> 60 years), prior thrombosis, and the presence of the JAK2V617F mutation have been reported as substantial risk factors for predicting MPN-associated thrombosis [71].

The association between IVCT and MPN has been addressed. One study reported no evidence of this relationship, based on its retrospective analytic results showing that no MPN or JAK-2 V617F mutation was identified in a cohort of 40 enrolled IVCT patients [10]. However, we advocated different viewpoints by proposing that IVCT can be associated with MPN, as evidenced from our ET patient presenting with IVCT and from other previous observations [72, 73]. It is likely that not only the well-known prothrombotic potential of MPN can predispose to IVCT, but splanchnic vein thrombosis occurring in some MPN patients may also contribute, including portal vein thrombosis, mesenteric vein thrombosis, splenic vein thrombosis, or BCS. Further investigation on this issue is required to define the relationship between IVCT and MPNs.

Furthermore, three of our IVCT patients were found to carry lupus anticoagulants, while none carried anticardiolipin antibodies. Pregnancy, surgery, and antithrombin deficiency were also identified, which is consistent with the etiology of IVCT reported in other studies (Table 3). In particular, no known risk factors were identified in one patient, and she was classified as having idiopathic/primary IVCT. However, it should be noted that our retrospective description could not ascertain any occult or obscure predisposing factors.

Finally, our report disclosed that an unretrieved IVC filter was a risk factor for IVCT, as supported by the evidence that two patients developed IVCT after an IVC filter was not removed. Our results highlighted the emerging risk of IVCT in Taiwan as a complication following IVC filter placement, which corresponds to the accumulating evidence gathered from other Asian countries with a reported relatively lower risk of VTE [74–76].

The effectiveness of IVC filters in preventing PE in VTE patients remains controversial and inconclusive. According to the PREPIC study [77], an IVC filter was shown to have the potential benefit of protecting against short-term PE (at 12 days, 1.1 % with PE in the filter group vs.4.8 % in the no-filter group, *p* = 0.03). However, using IVC filters had a higher risk of symptomatic DVT in the long term (at 2 years, 20.8 % in the filter group vs. 11.6 % in the no-filter group, *p* = 0.02), wherein the study showed no difference in the mortality rates during the 8-year follow-up period [43]. Furthermore, according to the 2020 American Society of Hematology (ASH) guidelines for the management of venous thromboembolism [78], increased mortality and incidence of subsequent DVT were observed in patients using IVC filters. These guidelines present an evaluation of seven systemic reviews and two randomized trials, indicating low confidence in the evidence for IVC filter efficacy. A similar conclusion of insufficient evidence supporting IVC filter effectiveness was also reported from the National Institutes for Health and Care Excellence (NICE) review in 2020 [79].

The 2016 American College of Chest Physicians (ACCP) guidelines recommend the use of an IVC filter in patients with acute proximal DVT but in whom anticoagulants are contraindicated, such as those with active uncontrollable bleeding [80]. It is also considered reasonable for IVC filters to be used in thromboembolic patients in whom anticoagulation is perceived to have failed [81]. However, the ACCP advises against the initial use of an IVC filter in addition to anticoagulants in patients with acute DVT of the leg, which is primarily due to the efficacy and safety concerns regarding IVC filters [80]. A similar recommendation was also advocated by the ASH guidelines [78]. Moreover, a low retrieval rate after the placement of retrievable filters further aggravates real-world adverse event-related conditions [82].

Filter-related thromboembolism is a complicated process, which may be influenced by various filter types and designs, patient-specific underlying conditions, such as pregnancy or malignancy, and the intrinsic thrombogenicity of the device. This thrombogenicity is possible since a filter thrombus can be formed from the entrapped emboli within the filter. Furthermore, the filter could induce thrombus formation, as it is a foreign body. A thrombus could also be formed by DVTextension from the iliac and lower extremities veins. In our opinion, IVC filters should be implanted judiciously under absolute indications while the filter device application in clinical thrombotic conditions without approved indications, such as prophylactic use in patients without a history of PE, is strongly discouraged. Additionally, an implanted IVC filter must be retrieved whenever possible.

## Conclusions

IVCT accounts for a substantial minority of DVT patients, although it is rare and liable to be underestimated. In accordance with venous thromboembolism, IVCT can be classified as either idiopathic/primary or provoked/secondary thrombosis, and various predisposing factors have been identified, including congenital IVC anomalies, malignancy, venous outflow obstruction/compression scenarios, such as BCS or May-Thurner syndrome, as well as hereditary thrombophilia, acquired APS especially lupus anticoagulants, and other risk factors for VTE. IVC filter-related complication has become an emerging cause of IVCT, which was also highlighted in our case series. Acknowledgement of these clinical high-risk features, as well as the relevant presentations, such as bilateral lower extremity swelling, lower back/pelvic pain, scrotum swelling, or PE, would help increase physicians’ awareness of IVCT. Anticoagulant therapy is the cornerstone of IVCT treatment after diagnosis, while CDT strategies might offer additional advantages.

## Data Availability

All data generated or analyzed during this study are included in this published article.

## Abbreviations

ACCP: American College of Chest Physicians
ASH: American Society of Hematology
BCS: Budd-Chiari syndrome
COVID-19: Coronavirus-2019
CDT: Catheter-directed thrombolysis
DVT: Deep venous thrombosis
ET: Essential thrombocythemia
FDA: Food and Drug Administration
IVC: Inferior vena cava
IVCT: Inferior vena cava thrombosis
MPN: Myeloproliferative neoplasm
NICE: National Institutes for Health and Care Excellence
PE: Pulmonary embolism
VTE: Venous thromboembolism
